# Acid-sensing ion channel 1a exacerbates renal ischemia–reperfusion injury through the NF-κB/NLRP3 inflammasome pathway

**DOI:** 10.1007/s00109-023-02330-7

**Published:** 2023-05-29

**Authors:** Yan Yang, Shi Jin, Jian Zhang, Weize Chen, Yufei Lu, Jun Chen, Zhixin Yan, Bo Shen, Yichun Ning, Yiqin Shi, Jing Chen, Jialin Wang, Sujuan Xu, Ping Jia, Jie Teng, Yi Fang, Nana Song, Xiaoqiang Ding

**Affiliations:** 1 Department of Nephrology, Zhongshan Hospital, Fudan University; Shanghai Medical Center of Kidney; Shanghai Institute of Kidney and Dialysis; Shanghai Key Laboratory of Kidney and Blood Purification; Hemodialysis quality control center of Shanghai, Shanghai, 200032 China; 2 Department of Pathology, Changzheng Hospital, Naval Military Medical University, Shanghai, China; 3 Fudan Zhangjiang Institute, Shanghai, China

**Keywords:** Acid-sensing ion channel 1a, NF-κB, NLRP3 inflammasome, Ischemia-reperfusion, Acute kidney injury

## Abstract

**Abstract:**

Ischemia-reperfusion injury (IRI) is the main cause of acute kidney injury (AKI), and there is no effective therapy. Microenvironmental acidification is generally observed in ischemic tissues. Acid-sensing ion channel 1a (ASIC1a) can be activated by a decrease in extracellular pH which mediates neuronal IRI. Our previous study demonstrated that, ASIC1a inhibition alleviates renal IRI. However, the underlying mechanisms have not been fully elucidated. In this study, we determined that renal tubule-specific deletion of ASIC1a in mice (ASIC1a^fl/fl^/CDH16^cre^) attenuated renal IRI, and reduced the expression of NLRP3, ASC, cleaved-caspase-1, GSDMD-N, and IL-1β. Consistent with these in vivo results, inhibition of ASIC1a by the specific inhibitor PcTx-1 protected HK-2 cells from hypoxia/reoxygenation (H/R) injury, and suppressed H/R-induced NLRP3 inflammasome activation. Mechanistically, the activation of ASIC1a by either IRI or H/R induced the phosphorylation of NF-κB p65, which translocates to the nucleus and promotes the transcription of NLRP3 and pro-IL-1β. Blocking NF-κB by treatment with BAY 11-7082 validated the roles of H/R and acidosis in NLRP3 inflammasome activation. This further confirmed that ASIC1a promotes NLRP3 inflammasome activation, which requires the NF-κB pathway. In conclusion, our study suggests that ASIC1a contributes to renal IRI by affecting the NF-κB/NLRP3 inflammasome pathway. Therefore, ASIC1a may be a potential therapeutic target for AKI.

**Key messages:**

- Knockout of ASIC1a attenuated renal ischemia-reperfusion injury.
- ASIC1a promoted the NF-κB pathway and NLRP3 inflammasome activation.
- Inhibition of the NF-κB mitigated the NLRP3 inflammasome activation induced by ASIC1a.

## Introduction

Acute kidney injury (AKI) is characterized by an abrupt decrease in kidney function over hours to days. This disorder occurs in approximately 20% of hospitalized patients [1]. Ischemia–reperfusion injury (IRI), which manifests as acute tubular necrosis, is the main cause of AKI [2]. Currently, there is no proven treatment to alleviate injury or enhance speed of recovery for IRI [3]. Despite advances in therapy, AKI has a high mortality rate, severe sequelae, and imposes a serious burden on public health resources.

Acid–base homeostasis is critical for maintaining normal physiological functions [4]. Microenvironmental acidification is generally observed in ischemic tissues. The pH value of kidney tissue and culture medium could drop from 7.4 to 6.5, or even lower, after ischemia and hypoxia treatment [5, 6]. Acid-sensing ion channels (ASICs) belong to the H^+^-gated Na^+^ channel family and are activated by extracellular acidosis [7]. In mammals, six ASIC subtypes, encoded by four genes, have been identified: ASIC1a, ASIC1b, ASIC2a, ASIC2b, ASIC3, and ASIC4. ASIC1a is the only channel permeable to Ca^2+^, in addition to Na^+^, and is highly proton sensitive. These characteristics lead to ASIC1a playing a crucial role in the development of many diseases such as stroke, liver fibrosis, and rheumatoid arthritis [8–10]. However, it was recently discovered that ASIC1a mediates acidic injury independent of its ion-conducting function [11]. Conformational changes in ASIC1a induced by acidosis lead to phosphorylation of downstream proteins such as receptor-interacting serine/threonine protein kinase 1 and the transcription factor nuclear factor-κB (NF-κB) [12, 13]. Our group previously demonstrated that ASIC1a is distributed in the renal tubule and that pharmacological inhibition of ASIC1a could protect the kidney against IRI [14]. However, the specific mechanism through which ASIC1a causes renal IRI remains unclear.

Inflammasomes that respond to microbial infections and cellular damage are vital components of the innate immune system. The nucleotide-binding oligomerization domain-like receptor family pyrin domain containing 3 (NLRP3) inflammasome has been widely investigated because of its involvement in IRI in organs such as the brain, heart, and testis [15–17]. It has been reported that NLRP3 is upregulated in renal IRI, and that NLRP3 deficiency attenuates renal dysfunction [18, 19]. The NLRP3 inflammasome is a multi-protein complex composed of NLRP3, apoptosis-associated speck-like protein containing a caspase recruitment domain (ASC), and pro-caspase-1. The assembly of the complex triggers pro-caspase-1 self-cleavage into active caspase-1, which converts the cytokine precursor pro-IL-1β into mature and secreted IL-1β. Gasdermin D (GSDMD) is also cleaved by caspase-1 to generate the N-terminal fragment (GSDMD-N), resulting in pyroptosis. The transcription of NLRP3 and pro-IL-1β is induced by activation of NF-κB [20, 21]. ASIC1a is involved in NF-κB transcriptional activity in endplate chondrocytes [13]. ASIC1a contributes to NLRP1 inflammasome activation in acidic neuronal injury [22]. Meanwhile, the expression of ASIC1 in the kidney tissue of Henoch–Schönlein purpura nephritis was positively correlated with renal inflammation severity [23]. However, whether ASIC1a regulates NLRP3 inflammasome activity, and the mechanisms underlying renal IRI remain unclear.

In this study, we investigated the effect of ASIC1a on NLRP3 inflammasome activity in renal IRI. We demonstrate that ASIC1a activates the NLRP3 inflammasome through NF-κB to promote renal IRI. Therefore, ASIC1a may serve as a potential therapeutic target for AKI.

## Materials and methods

### Animals

The Cre/loxP recombination system was used to generate ASIC1a^fl/fl^/CDH16^cre^mice. Mice that were maintained on the C57BL/6 background with the ASIC1a floxed allele were crossed with mice that expressed Cre recombinase under the cadherin 16 promoter (CDH16^Cre^). The ASIC1a^fl/fl^ and wild-type alleles were detected using the following primers: 5’- GTGTTGTTTGCTTCTGGCCG-3’ and 5’- CAGATGACAGTTTGCAGGCC-3’, which generated a 442 bp product in the floxed allele and a 281 bp product in the wild-type allele. The CDH16-Cre transgene was detected using primers 5’- GCAGATCTGGCTCTCCAAAG-3’ and 5’- AGGCAAATTTTGGTGTACGG-3’, which amplified a 420 bp fragment. The internal positive control was detected using the primers 5’- CAAATGTTGCTTGTCTGGTG-3’ and 5’- GTCAGTCGAGTGCACAGTTT-3’, which amplified a 200 bp fragment. C57BL/6 wild-type (WT) mice and floxed without CDH16-Cre (ASIC1a^fl/fl^) mice served as the control group for tubule-specific ASIC1a deletion (ASIC1a^fl/fl^/CDH16^cre^) mice. Mice were housed in an acclimatized room and allowed free access to food and water. All experimental procedures were approved by the Institutional Animal Care and Use Committee of Fudan University.

### Renal IRI model in mouse

Renal ischemia AKI was induced in 8-week-old male mice [14]. Briefly, following treatment with 4% phenobarbitone (10 mL kg^−1^ body weight), bilateral renal pedicles were exposed and clamped to induce 35 min of ischemia. The atraumatic clamps were then released. The sham-operated group underwent the same procedure without clamping. The body temperature of mice was maintained at 37 °C. The kidneys and blood were harvested after 24 h reperfusion.

### Renal function

Blood was collected by cardiac puncture. Serum creatinine levels were determined using a Quantichrom Creatinine Assay kit (BioAssay Systems, Hayward, CA, USA).

### Histopathological examinations

Paraffin-embedded 5-μm kidney sections were stained with periodic acid-Schiff (PAS). Histological injury scores were evaluated by light microscopy in a blinded manner. Severity was determined in a semi-quantitative manner by the percentage of tubules manifesting epithelial necrosis, loss of the brush border, cast formation, and tubular dilation. A five-point scale was used, as follows: 0, no injury; 1, less than 25%; 2, 25–50%; 3, 50–75%; and 4, more than 75%.

### Real-time quantitative PCR

Total RNA was extracted with TRIzol reagent. First-strand cDNA was synthesized by reverse transcription. Quantitative PCR was performed with SYBR-green. Primers are described in Table 1. Relative levels of mRNA expression were normalized to GAPDH expression for each gene.

**Table 1:** Primer sets used for real‐time PCR

**Gene**	**Sense Primer (5**’**-3**’**)**	**Antisense Primer (5**’**-3**’**)**
mouse KIM-1	ATCCCATACTCCTACAGACT	CCAACATAGAAGCCCTTA
mouse NGAL	AAGGCAGCTTTACGATGT	TGGTTGTAGTCCGTGGTG
mouse GAPDH	AGGTCGGTGTGAACGGATTTG	GGGGTCGTTGATGGCAACA

### Determination of ROS

Dihydroethidium (DHE) staining was used to characterize reactive oxygen species (ROS) production. Cryosections were incubated with 10 μM DHE (red fluorescence) at 37 °C for 30 min followed by staining with DAPI (blue fluorescence), and then observed by confocal microscopy. For quantification, the fluorescent density was determined.

### Flow cytometry

Kidneys were collected to determine the neutrophil infiltration by flow cytometry. Briefly, kidney tissue was homogenized after digestion with collagenase/DNase I. Cell suspensions were incubated with fluorescently-labeled antibodies against Ly6G and CD45. Flow cytometry was then performed. Neutrophils were identified as Ly6G^+^ CD45^+^ cells. The result was expressed as fraction of Ly6G^+^ CD45^+^ cells among total CD45^+^ cells.

### Cell culture and treatments

The human kidney proximal tubular epithelial cell line (HK-2, ATCC) was incubated in a humidified atmosphere containing 5% CO_2_ at 37 °C. For hypoxia/reoxygenation (H/R), cells were cultured in incubator containing 1% O_2_, 94% N_2_, and 5% CO_2_ for 6 h and subsequently cultured in normoxic conditions with 21% O_2_ for 1 h. For the acidosis treatment, the pH of the medium was adjusted using an appropriate amount of HCl. Psalmotoxin-1 (PcTx-1; peptide institute, Japan) and BAY 11-7082 (Beyotime, Shanghai, China) were dissolved and diluted in ddH_2_O. The cells were treated with PcTx-1 or BAY11-7082 for 1 h prior to H/R or acidosis.

### Cell viability

HK-2 cells were seeded in 96-well plates at a density of 5 × 10^3^ cells per well. After treatment, the cells were incubated with the CCK-8 reagent (Dojindo, Kumamoto, Japan). The absorbance of CCK-8 was measured at 450 nm wavelength using a microplate reader.

### Western blotting

The protein samples were separated and transferred to a PVDF membrane for incubation with primary antibodies against NLRP3 (1:1000), ASC (1:400), caspase-1 (1:1000), GSDMD-N (1:1000), IL-1β (1:800), phosphorylated NF-κB p65 (p-NF-κB p65, 1:1000), NF-κB p65 (1:1000), and GAPDH (1:4000) at 4 °C overnight. Then the membranes were incubated with secondary antibodies. Protein band intensities were quantified using densitometry. All values were normalized to GAPDH and expressed as fold change relative to the control. Detailed information on the primary antibodies is given in Table 2.

**Table 2:** List of primary antibodies

**Primary Antibody**	**Company**	**Catalog**
NLRP3	Proteintech	19771-1-AP
	Abcam	ab214185
ASC	Cell Signaling Technology	67824
	Abcam	ab155970
Caspase-1	Abcam	ab207802
	Abcam	ab108362
GSDMD-N	Abcam	Ab210070
IL-1β	Cell Signaling Technology	12242
	ABclonal	A1112
phosphorylated NF-κB p65	Abcam	ab76302
NF-κB p65	Abcam	ab16502
GAPDH	Abcam	ab8245
ASIC1a	Alomone Labs	AGP-053
AQP1	Abcam	ab168387

### TUNEL assay

Apoptosis was detected using a TUNEL apoptosis assay kit (Beyotime, Shanghai, China). Briefly, 5-μm kidney sections were deparaffinized and rehydrated, or HK-2 cells were fixed. The samples were incubated with the TUNEL (green fluorescence) reagent mixture for 1 h at 37 °C, and the nuclei were stained with DAPI (blue fluorescence). We calculated six view fields per specimen under magnification × 400 in a blinded manner to the treatment. Cells expressing both green and blue fluorescence were considered as apoptotic cells. The number of apoptotic cells in each field was counted and the result was expressed as Number/VF.

### Immunohistochemistry

For NLRP3 immunohistochemistry, 5-μm kidney sections were incubated with antibody. Then the reactions were developed using an avidin–biotin-HRP complex immunodetection kit and photographed under a light microscope. Relative area (positive staining/background) of positive stained cells were calculated in six random sections from each animal under magnification × 200. The result was expressed as the percentage of positive area.

### Immunofluorescence

Immunofluorescence double staining was performed on frozen sections as previously described [14]. Briefly, slices were incubated with guinea pig anti-ASIC1a antibody (1:50) and rabbit anti-aquaporin 1 (AQP1, 1:100). Secondary antibodies were Alexa Fluor 488-conjugated donkey anti-guinea pig IgG (1:100) and Alexa Fluor 594-conjugated donkey anti-rabbit IgG (1:100). Visualization was under a confocal microscope.

For NF-κB p65 immunofluorescence, HK-2 cells were fixed, permeabilized and then blocked. The cells were incubated with rabbit anti-NF-κB p65 (1:1000), followed by Alexa Fluor 594-conjugated donkey anti-rabbit IgG (1:200), and counterstained with DAPI. Images were acquired using an inverted fluorescence microscope. Detailed information on the primary antibodies is given in Table 2.

### Statistical analysis

All values are expressed as mean ± standard deviation (SD) and were analyzed using SPSS. Data were analyzed by one-way ANOVA or two-way ANOVA with subsequent post hoc Student and Newman-Keul’s tests where applicable. Statistical significance was set at P < 0.05.

## Results

### Knockout of ASIC1a in the kidney epithelium attenuates I/R-induced kidney injury

**Fig. 1:**
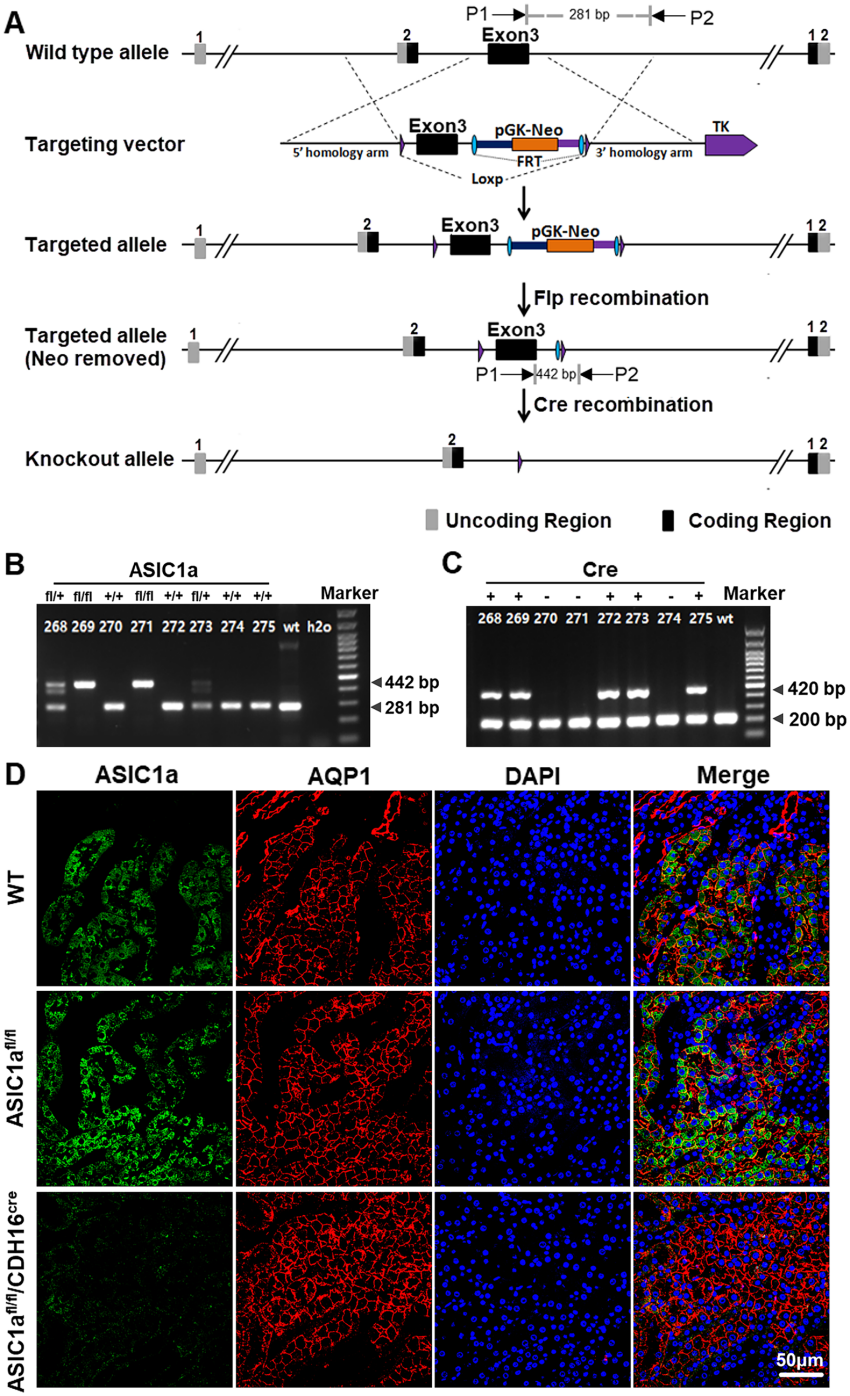
Generation of conditional renal tubular ASIC1a-knockout mice (ASIC1a^fl/fl^/CDH16^cre^). **A** Schematic diagram of constructing ASIC1a^fl/fl^/CDH16^cre^ mice; P1 and P2 were used for the PCR genotyping of exon 3 in the ASIC1a gene. **B** PCR genotyping of the floxed allele: primers P1 and P2 amplify a PCR product of 442 bp for the floxed allele and 281 bp for the wild-type allele, respectively. **C** PCR genotyping of CDH16^cre^: primers amplify a PCR product of 420 bp. **D** Representative images of co-immunostaining ASIC1a and AQP1. ASIC1a, acid-sensing ion channel 1a; CDH16, Cadherin 16; AQP1, aquaporin 1, proximal tubular cells marker; DAPI, 4,6-diamidino-2-phenylindole, for nuclei

We previously demonstrated that renal ischemia increases the expression of ASIC1a, and injection of the ASIC1a inhibitor PcTx-1 attenuates renal ischemic injury in vivo [14]. To further explore the role of ASIC1a in I/R-induced renal injury, we generated conditional renal tubular ASIC1a-knockout mice (ASIC1a^fl/fl^/CDH16^cre^) by crossing ASIC1a^fl/fl^ mice with CDH16-Cre mice (Fig. 1A). We confirmed ASIC1a^fl/fl^ or the wild-type allele gene (Fig. 1A, B) and the CDH16-Cre transgene by PCR (Fig. 1C). ASIC1a is mainly found in proximal tubule cells, the major site of renal IRI. Therefore, we verified deletion of ASIC1a from the proximal tubular cells by co-staining with the nephron segment-specific marker AQP1 (Fig. 1D).

**Fig. 2:**
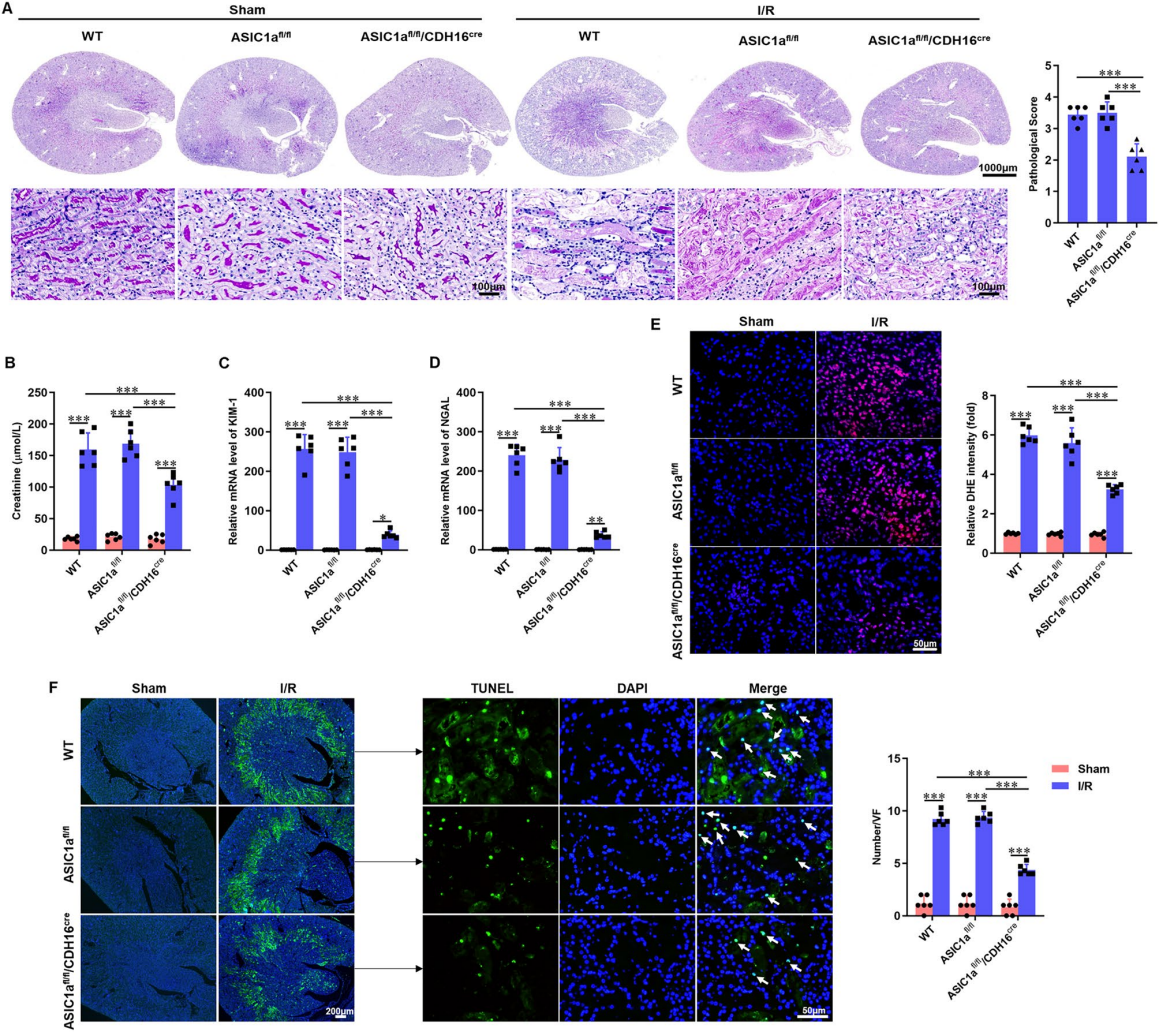
Knockout of ASIC1a in the kidney epithelium attenuates renal IRI. WT, ASIC1a^fl/fl^, and ASIC1a^fl/fl^/CDH16^cre^ mice were subjected to renal I/R. Twenty-four hours after reperfusion, kidney and serum samples were collected. **A** Typical visual field of PAS staining (left panel) and pathological score calculated from PAS staining (right panel). **B** Levels of serum creatinine. **C**,** D** Quantification of kidney KIM-1 and NGAL (markers of renal tubular damage) mRNA levels by qPCR. **E** Typical image of DHE staining (left panel) and quantification of DHE fluorescence intensity (right panel). **F** Typical image of TUNEL staining (left panel) and the number of TUNEL-positive cells in the photographic area (right panel); TUNEL-positive cells indicated by white arrows. *P < 0.05, **P < 0.01, ***P < 0.001, n = 6

Importantly, ASIC1a deficiency did not affect normal renal function. Renal I/R resulted in kidney tubular necrosis, cast formation, tubular dilation, and loss of the brush border at 24 h after reperfusion. Compared to WT and ASIC1a^fl/fl^ mice, ASIC1a^fl/fl^/CDH16^cre^ mice exhibited less tubular damage and a markedly lower mean pathological score (Fig. 2A). Additionally, ASIC1a^fl/fl^/CDH16^cre^ mice showed a corresponding reduction in serum creatinine levels and mRNA expression of KIM-1 and NGAL (Fig. 2B–D). Oxidative stress and apoptosis are important in the pathophysiology of renal IRI. We detected DHE to reflect the ROS level in the kidney tissue. Quantification analysis demonstrated that I/R induced DHE signals and ASIC1a^fl/fl^/CDH16^cre^ mice had significantly weaker DHE signals than WT and ASIC1a^fl/fl^ mice (Fig. 2E). We also examined whether ASIC1a modulated I/R-mediated apoptosis using TUNEL staining. No detectable apoptotic nuclei were observed in sham-operated mice. ASIC1a^fl/fl^/CDH16^cre^ mice displayed reduced renal apoptosis with fewer TUNEL-positive cells (Fig. 2F). These results indicated that ASIC1a deficiency could protect against IRI, and suggested that ASIC1a exacerbates renal IRI.

### Knockout of ASIC1a in the kidney epithelium reduces IRI-induced NLRP3 inflammasome activation by inhibiting NF-κB

**Fig. 3:**
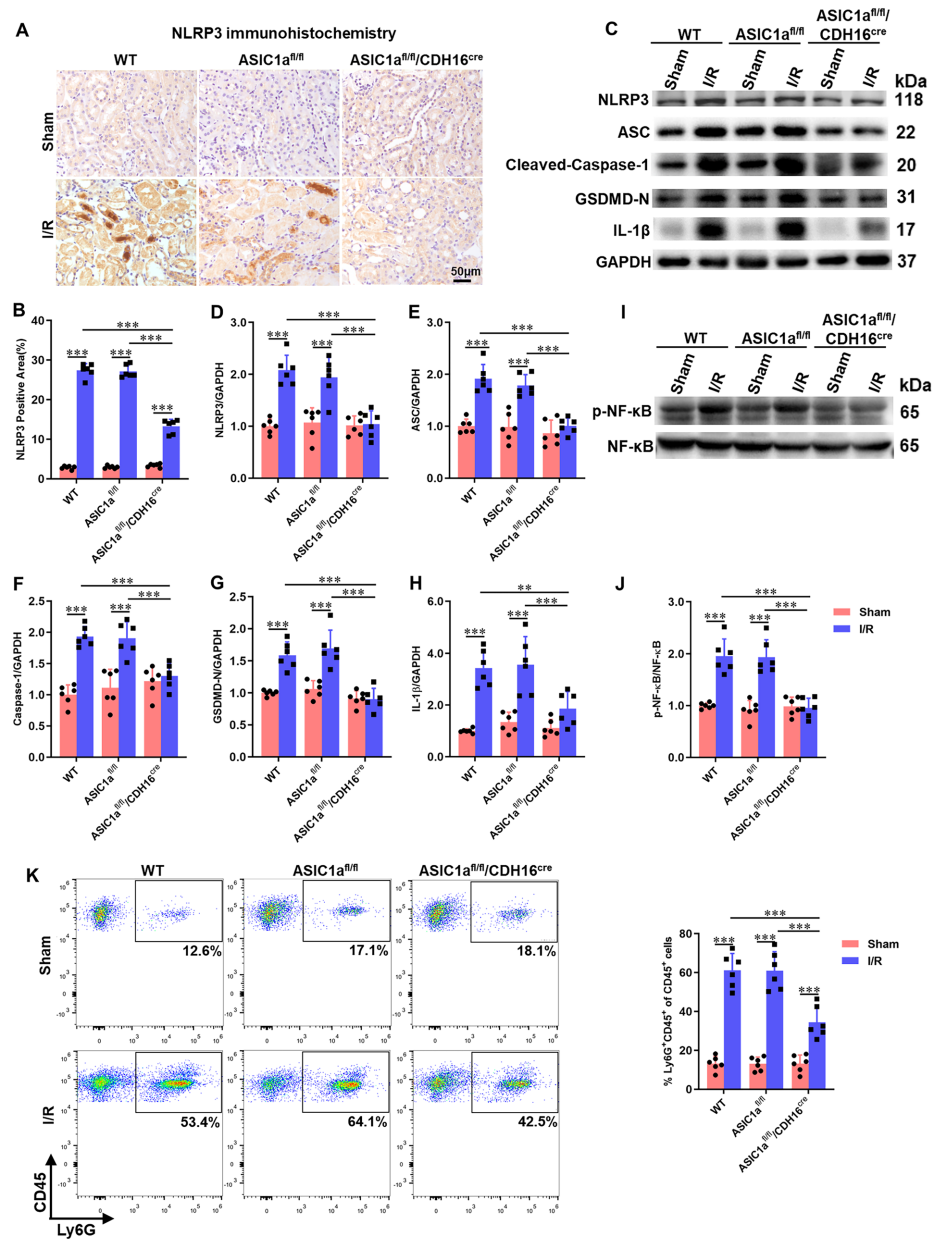
Knockout of ASIC1a in the kidney epithelium reduces IRI-induced NLRP3 inflammasome activation by inhibiting NF-κB. **A** Immunohistochemistry analysis of NLRP3 expression in kidney tissues. **B** Percentage of positive staining for the molecule measured relative to the photographic area. **C**–**J** Western blot and densitometry analysis of NLRP3, ASC, cleaved-caspase-1 p20, GSDMD-N, IL-1β, p-NF-κB p65, and NF-κB p65 expression in kidney tissues. **K** Fraction of Ly6G^+^ CD45^+^ cells among total CD45^+^ cells. *P < 0.05, **P < 0.01, ***P < 0.001, n = 6

The NLRP3 inflammasome, a component of innate immunity, plays a critical role in renal IRI. The effect of ASIC1a on IRI-induced NLRP3 inflammasome activation was thus investigated. The expression of NLRP3 was analyzed using immunohistochemistry and western blotting. ASIC1a deficiency significantly diminished NLRP3 expression (Fig. 3A, B). Western blot analysis confirmed that renal IRI induced the activation of the NLRP3 inflammasome, as evidenced by increased expression of NLRP3, ASC, cleaved-caspase-1 p20, GSDMD-N, and IL-1β, whereas knockout of ASIC1a reduced the activation of the NLRP3 inflammasome (Fig. 3C–H). It has been reported that NF-κB can activate the transcription of NLRP3 [20]. Western blotting results showed that I/R increased the protein expression of p-NF-κB p65, while ASIC1a knockout decreased its expression (Fig. 3I, J). We furtherly assessed neutrophil infiltration in whole kidney tissue using flow cytometry. We found that I/R induced accumulation of neutrophil (Ly6G^+^ CD45^+^ cells) in the kidney and knockout of ASIC1a inhibited neutrophil infiltration, shown by lower neutrophil (Ly6G^+^ CD45^+^ cells) fraction (Fig. 3K). Taken together, these results indicated that deletion of ASIC1a reduced I/R-induced inflammatory reaction in the kidney.

### Inhibition of ASIC1a by PcTx-1 protects HK-2 cells from H/R injury and reduces H/R-induced NLRP3 inflammasome activation

**Fig. 4:**
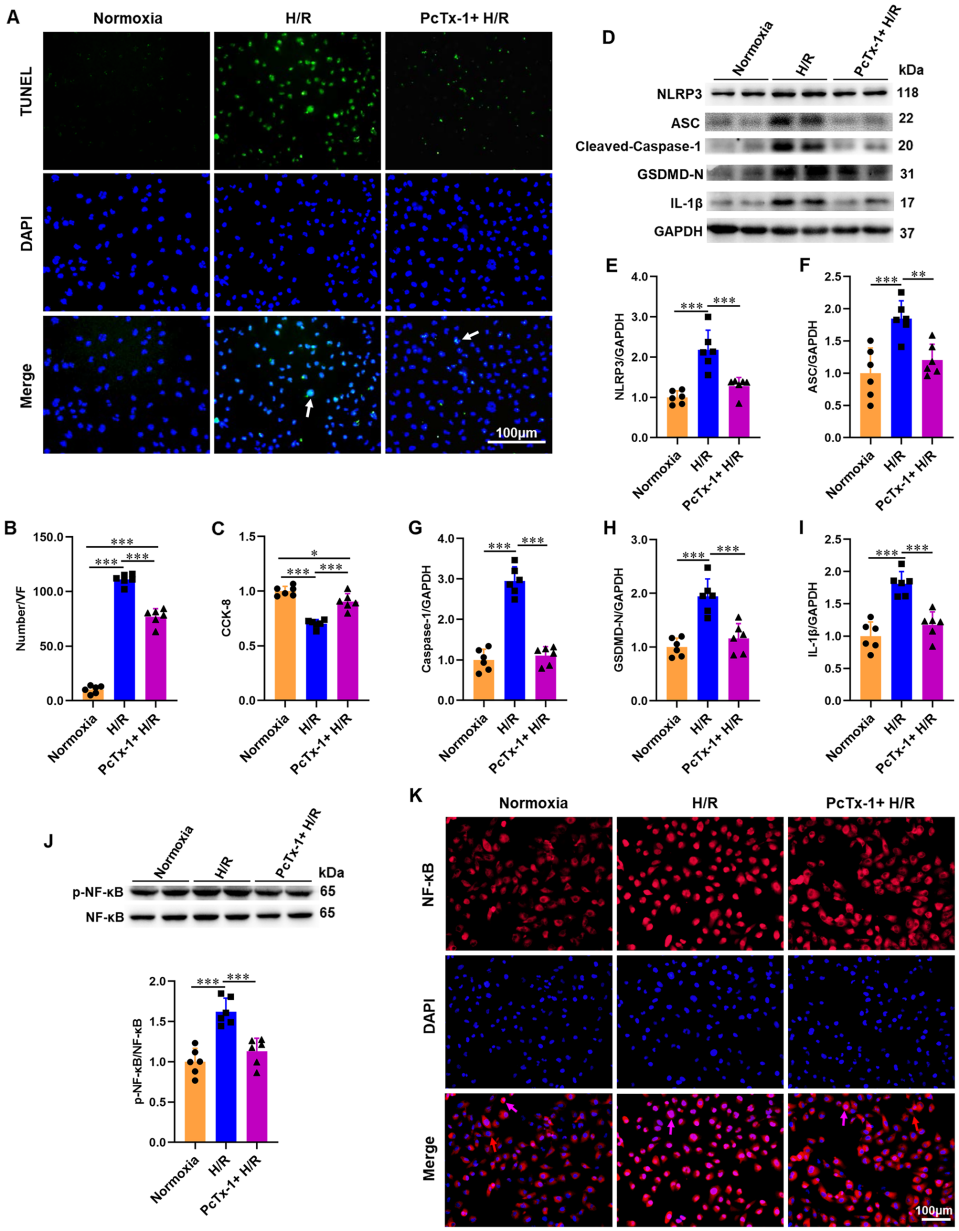
Inhibition of ASIC1a protects HK-2 cells from H/R injury and reduces H/R-induced NLRP3 inflammasome and NF-κB activation. PcTx-1 (25 ng mL^−1^) was administered to HK-2 cells before H/R treatment. **A** Typical image of apoptosis measured by TUNEL in HK-2 cells. **B** The number of TUNEL-positive cells in the photographic area. **C** The cell viability of HK-2 cells assessed by CCK-8 assay. **D**–**J** Western blot and densitometry analysis of NLRP3, ASC, cleaved-caspase-1 p20, GSDMD-N, IL-1β, p-NF-κB p65, and NF-κB p65 expression in HK-2 cells. **K** Typical image of the NF-κB p65 nuclear translocation in HK-2 cells; nucleus NF-κB p65 indicated by purple arrows; cytoplasm NF-κB p65 indicated by red arrows. *P < 0.05, **P < 0.01, ***P < 0.001, n = 6

To confirm the effect of ASIC1a on ischemic renal injury, PcTx-1 (25 ng ml^−1^) was applied to block ASIC1a in HK-2 cells before H/R treatment. Apoptosis of HK-2 cells was measured using TUNEL staining, and cell viability was assessed using the CCK-8 assay. H/R significantly induced apoptosis and decreased cell viability compared to control cells. Pretreatment with PcTx-1 attenuated apoptosis and improved cell viability (Fig. 4A–C).

To further demonstrate the effect of ASIC1a on NLRP3 inflammasome activation, we treated HK-2 cells with PcTx-1 before H/R stimulation and evaluated NLRP3 inflammasome activation. The expression levels of NLRP3, ASC, cleaved-caspase-1 p20, GSDMD-N, and IL-1β in HK-2 cells were measured using western blotting, and all were found to increase with H/R and decrease with PcTx-1. Thus, H/R treatment promoted NLRP3 inflammasome activation, and blocking ASIC1a by PcTx-1 attenuated this activation (Fig. 4D–I).

### Inhibition of ASIC1a decreases H/R-induced NF-κB signaling activation

ASIC1a regulates NF-κB transcriptional activity in endplate chondrocytes [13]. To elucidate the role of ASIC1a in the NF-κB signaling pathway in HK-2 cells, the expression of key molecules in the NF-κB family, including phospho-NF-κB p65 (p-NF-κB p65) and NF-κB p65, was tested in PcTx-1 pretreated HK-2 cells. Western blotting showed that H/R increased the protein expression of p-NF-κB p65, whereas inhibition of ASIC1a decreased the expression of p-NF-κB p65 (Fig. 4J). The translocation of NF-κB p65 from the cytoplasm to the nucleus is a prerequisite for the activation of the NF-κB pathway. Immunofluorescence showed that nuclear translocation of NF-κB p65 was increased by H/R and substantially repressed by PcTx-1 in HK-2 cells (Fig. 4K), indicating that ASIC1a could regulate NF-κB signaling activation.

### Stimulation of ASIC1a promotes activation of NLRP3 inflammasome through the NF-κB pathway

**Fig. 5:**
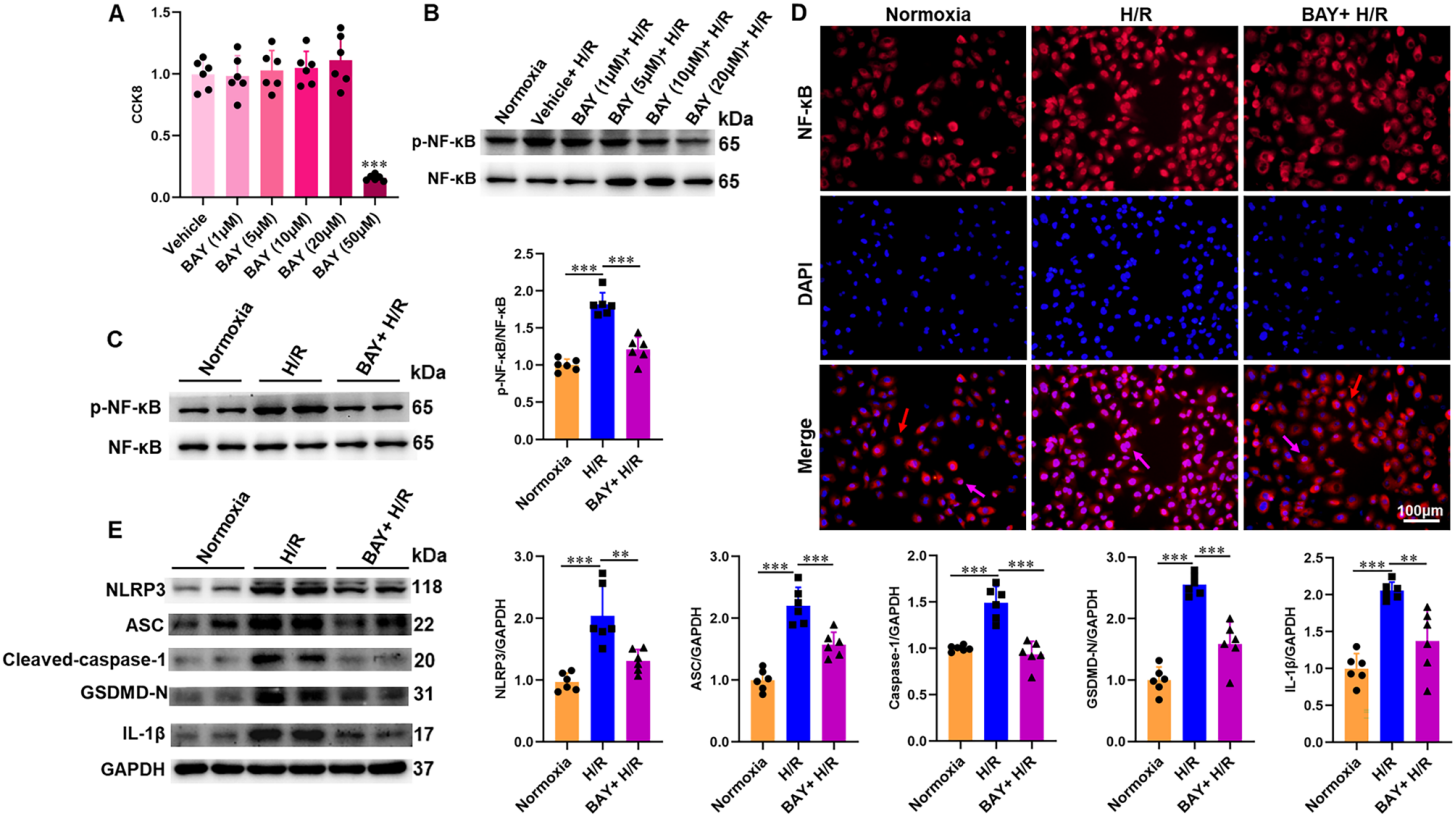
ASIC1a promotes H/R-induced NLRP3 inflammasome through the NF-κB pathway. HK-2 cells were pretreated with BAY 11-7082 before H/R treatment. **A** CCK-8 assay indicated that a high concentration (50 μM) of BAY 11-7082 was toxic to HK-2 cells. **B** Western blot showed that BAY 11-7082 at 10 μM significantly reduced expression of p-NF-κB p65. **C** Western blot and densitometry analysis of p-NF-κB p65 and NF-κB p65 expression in HK-2 cells. **D** Typical image of the NF-κB p65 nuclear translocation in HK-2 cells; nucleus NF-κB p65 indicated by purple arrows; cytoplasm NF-κB p65 indicated by red arrows. **E** Western blot and densitometry analysis of NLRP3, ASC, cleaved-caspase-1 p20, GSDMD-N, and IL-1β expression in HK-2 cells. *P < 0.05, **P < 0.01, ***P < 0.001, n = 6

To validate the role of NF-κB in H/R-induced NLRP3 inflammasome activation, a specific NF-κB inhibitor BAY 11-7082 was administered prior to H/R treatment in HK-2 cells. The dose of BAY 11-7082 was determined using a dose curve (1, 5, 10, 20, and 50 μM). The CCK-8 assay showed that treatment with BAY 11-7082 (1, 5, 10, and 20 μM) for 6 h had no effect on the viability of HK-2 cells, whereas 50 μM BAY 11-7082 sharply decreased cell viability (Fig. 5A). We found that BAY 11-7082 effectively inhibited NF-κB at 10 and 20 μM **(**Fig. 5B). Therefore, a dose of 10 μM was chosen. The protein expression of p-NF-κB p65, together with the nuclear translocation of NF-κB p65, was significantly suppressed in the BAY 11-7082-pretreated groups (Fig. 5C, D). BAY 11-7082 blocked NLRP3 inflammasome activation, as demonstrated by Western blot analysis of NLRP3, ASC, cleaved-caspase-1 p20, GSDMD-N, and IL-1β (Fig. 5E).

**Fig. 6:**
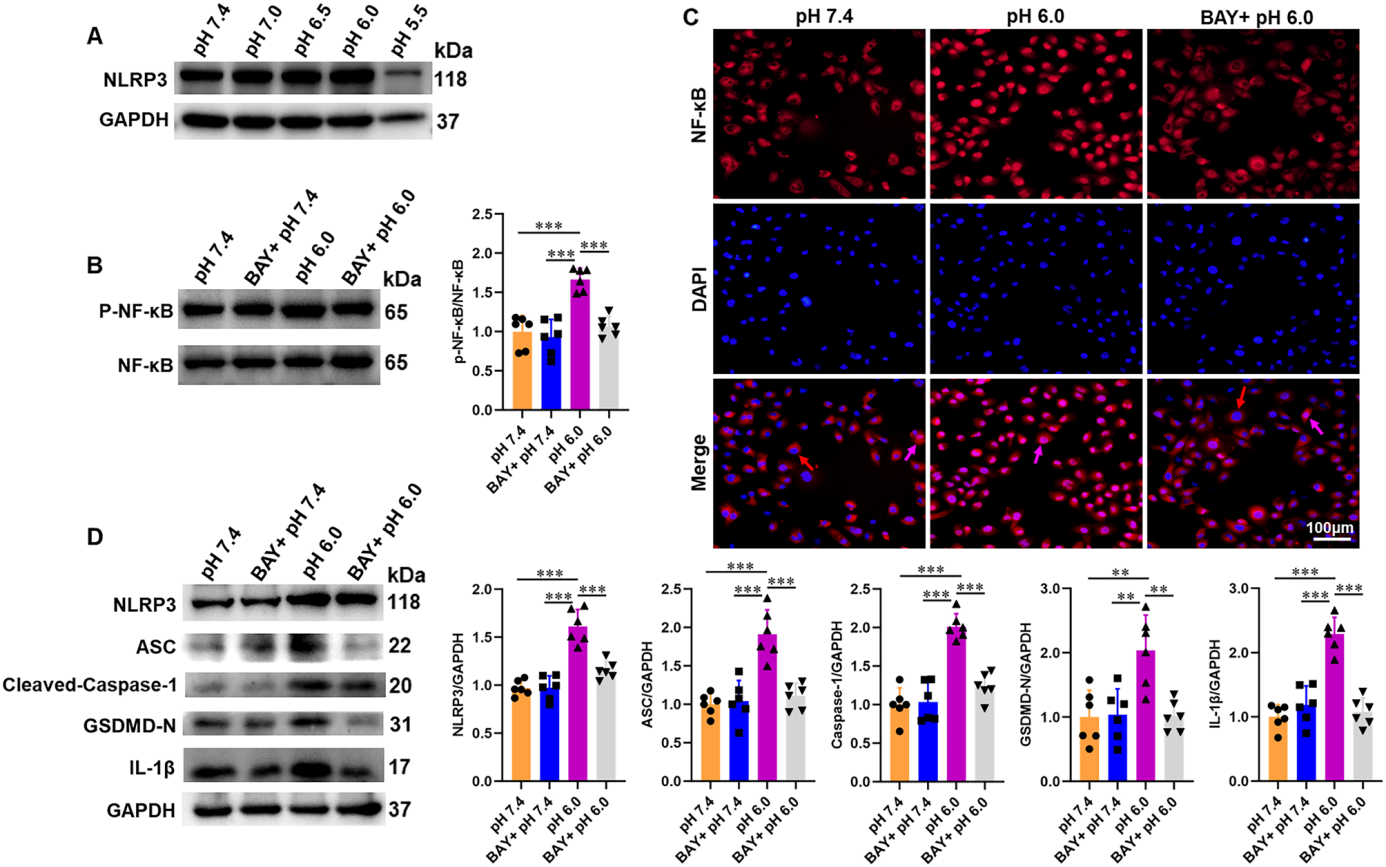
ASIC1a promotes the acidosis-induced NLRP3 inflammasome through the NF-κB pathway. HK-2 cells were pretreated with BAY 11-7082 before acidifying the medium. **A** Western blot of NLRP3 expression in HK-2 cells stimulated with different pH’s. **B** Western blot and densitometry analysis of p-NF-κB p65 and NF-κB p65 expression in HK-2 cells. **C** Typical image of NF-κB p65 nuclear translocation in HK-2 cells; nucleus NF-κB p65 indicated by purple arrows; cytoplasmic NF-κB p65 indicated by red arrows. **D** Western blot and densitometry analysis of NLRP3, ASC, cleaved-caspase-1 p20, GSDMD-N, and IL-1β expression in HK-2 cells. *P < 0.05, **P < 0.01, ***P < 0.001, n = 6

As a ligand of ASIC1a, a rise in extracellular H^+^ (acidification) could stimulate ASIC1a. Hypoxia usually leads to progressive acidification of the cell culture medium. To confirm the role of ASIC1a in the NF-κB/NLRP3 inflammasome pathway, ASIC1a was activated via extracellular acidification. We found that lowering the pH of the culture medium to 6.0 induced NLRP3 activation to a significant extent (Fig. 6A). BAY 11-7082 abolished acidosis-induced NF-κB activation (Fig. 6B, C), which was accompanied by a decreased protein expression of NLRP3, ASC, cleaved-caspase-1 p20, GSDMD-N, and IL-1β (Fig. 6D).

## Discussion

**Fig. 7:**
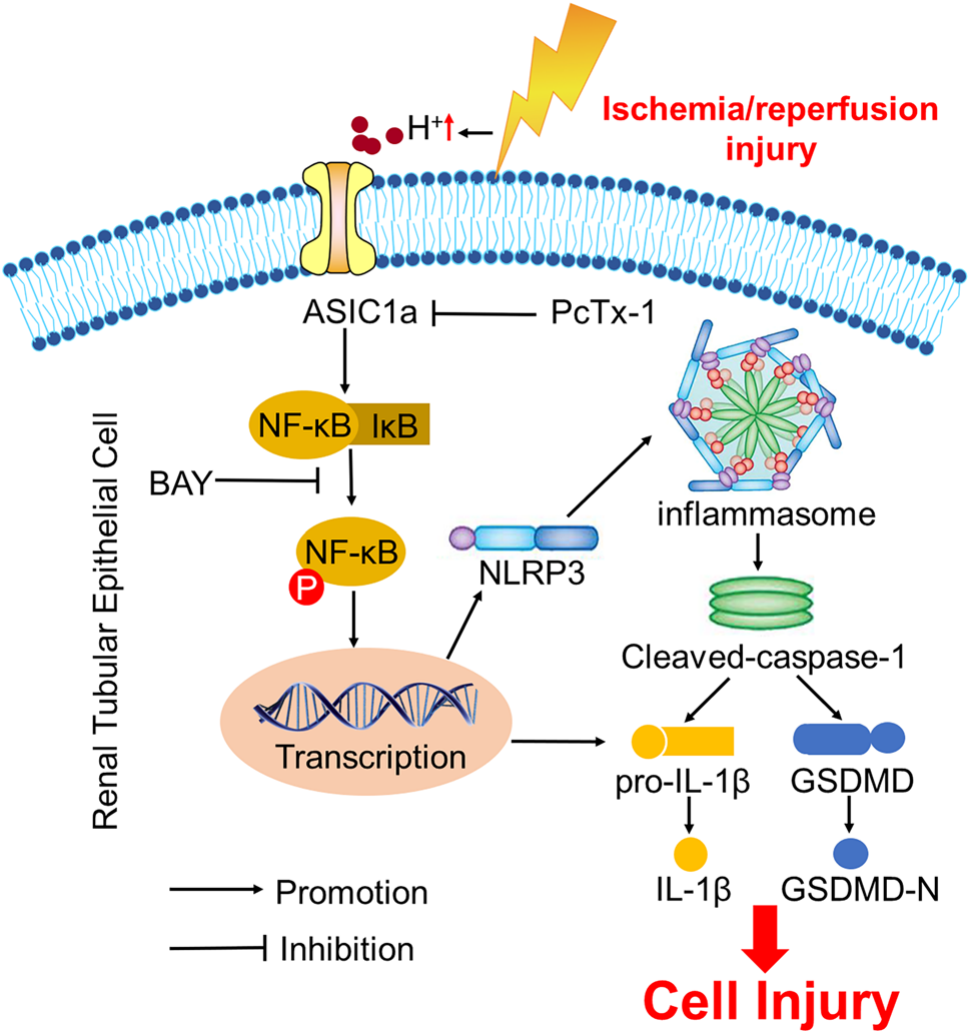
A schematic representation of the proposed model: ASIC1a is involved in renal IRI via the NF-κB/NLRP3 inflammasome. ASIC1a is activated by ischemia-induced extracellular acidosis and subsequently causes activation of NF-κB. NF-κB upregulates the expression of NLRP3 and IL-1β precursor and promotes activation of the NLRP3 inflammasome, which results in renal IRI. Inhibition of NF-κB pathway mitigates the activating effect of ASIC1a toward the NLRP3 inflammasome. Inhibiting ASIC1a by gene deletion or chemical agent protects against renal IRI. ASIC1a mediating the NLRP3 inflammasome activation by NF-κB pathway is critical in AKI. ASIC1a may represent a potential therapeutic target for AKI

IRI is the primary cause of AKI. Ischemic injury leads to microenvironmental acidification, which is associated with the activation of inflammation. In the present study, we found that ASIC1a, as a sensor of extracellular acidification, plays a major role in AKI, as evidenced by attenuated renal ischemia injury in ASIC1a^fl/fl^/CDH16^cre^ mice. The NLRP3 inflammasome is a cytosolic danger-recognition platform that responds to cellular damage. Our results indicated that the NLRP3 inflammasome is activated by ischemic injury in vivo and in vitro, which is blunted by the deficiency or pharmacological inhibition of ASIC1a. Additionally, ASIC1a activation promotes the NF-κB pathway. Inhibition of the NF-κB pathway mitigated the induction by ASIC1a of the NLRP3 inflammasome. ASIC1a-mediated NLRP3 inflammasome activation via the NF-κB pathway played an important role in AKI (Fig. 7).

Accumulating evidence has demonstrated that ASIC1a participates in the pathophysiology of various diseases such as cerebral infarction, rheumatoid arthritis and liver fibrosis [24–26]. In patients with Henoch-Schönlein purpura nephritis, it was reported that ASIC1a is highly expressed in renal tubular cells, and the protein level of ASIC1a is positively associated with the severity of renal injury [23]. Renal IRI, a predominant cause of AKI, often occurs during hemorrhagic shock, kidney transplantation, and cardiac surgery. Metabolic acidosis resulting from anaerobic glycolysis is a fundamental mechanism underlying IRI. In the brain, ischemia can reduce tissue pH to 6.5–6.0, and in the heart, the pH of the ischemic area can decrease to 6.7–6.8 [27, 28]. In the kidney, the pH can fall to 6.5 within five minutes following ischemia. Similar to ischemia in vivo, hypoxia can also cause acidification of the cell culture medium in vitro. It was reported that the pH value of cell culture medium decreased to 6.4–6.5 after 4 h of hypoxia treatment [5, 6]. ASIC1a has a high sensitivity to protons, which can be activated when the extracellular pH falls below 7.0 [29]. Thus, ischemia- or hypoxia-induced microenvironmental acidification readily activates ASIC1a. Increasing evidence indicates that Ca^2+^-permeable ASIC1a is partly responsible for ischemic brain injury [30, 31]. Blockade of ASIC1a prolongs the neuroprotective window, suggesting that ASIC1a is a potential novel therapeutic target for ischemic stroke [32]. Our previous studies have demonstrated that ASIC1a is expressed in renal tubule cells. Ischemia increased the expression of ASIC1a both in vivo and in vitro. Moreover, the ASIC1a specific inhibitor PcTx-1 alleviated renal IRI [14]. However, its exact mechanism of action remained unclear.

To verify the role of ASIC1a in renal IRI, we established a renal tubular conditional ASIC1a knockout mouse line (ASIC1a^fl/fl^/CDH16^cre^). Compared to WT and ASIC1a^fl/fl^ mice, we found that renal tubular-specific knockout ASIC1a mice exhibited less tubular damage and a corresponding reduction in serum creatinine and mRNA expression of KIM-1 and NGAL. Additionally, we found that blocking ASIC1a by PcTx-1 inhibited the decrease in cell viability caused by H/R treatment in vitro. Oxidative stress and apoptosis both take part in the development of renal IRI [33]. Knockout of ASIC1a attenuated the I/R-induced increase in DHE signals and TUNEL-positive cells. These results strongly suggested that ASIC1a is involved in renal IRI.

The NLRP3 inflammasome is a cytosolic danger-recognition platform that responds to microbial infection and cellular damage, including ischemic tissue injury [34]. Although the mechanism of NLRP3 inflammasome activation is poorly understood, substantial evidence indicates that the NLRP3 inflammasome-driven inflammatory response is crucial to the pathophysiology of renal IRI. NLRP3 deficiency reduces renal injury and mortality through anti-inflammatory and anti-apoptotic effects [19, 35–37]. Moreover, NLRP3 knockout can facilitate renal repair after IRI by promoting the proliferation of renal tubular epithelial cells [18]. It has been reported that an acidic extracellular pH triggers NLRP3 inflammasome activation and IL-1β secretion [38]. It has been reported that ASIC1a is expressed in inflammatory cells, such as macrophages and dendritic cells, and plays an important role in innate immunity [39, 40]. In addition, ASIC1a is expressed in inherent cells and regulates immune responses. For example, ASIC1a regulates nucleus pulposus cell pyroptosis via the NLRP3 inflammasome to promote intervertebral disc degeneration [41]. ASIC1a contributes to the activation of the NLRP3 inflammasome by upregulating intracellular Ca^2+^ in particular chondrocytes [42]. However, whether ASIC1a plays a role in renal IRI-induced NLRP3 inflammasome activation was unclear. In the current study, we observed that knocking out or blocking ASIC1a reduced renal IRI-induced upregulation of the NLRP3 inflammasome in vivo and in vitro. Our results indicated that stimulation of ASIC1a is involved in the activation of the NLRP3 inflammasome in renal IRI.

The transcription factor NF-κB is involved in the expression of multiple genes and controls many cellular processes, such as immune and inflammatory responses, cell proliferation, migration, and apoptosis [43]. NF-κB is a known activator of NLRP3, promoting the transcription of NLRP3 and IL-1β precursor. A variety of stimuli, including acidosis, cytokines, and oxidative stress, can induce the activation of NF-κB [44]. Several studies have shown that ASIC1a is critical for NF-κB activation. In tumorigenesis, ASIC1a is responsible for acidosis-induced NF-κB activation via the Akt and ERK pathways [45, 46]. In endplate chondrocytes, ASIC1a participates in matrix metabolism by regulating NF-κB activity under acidic conditions [13]. Activated NF-κB can in turn upregulate ASIC1a expression [47, 48]. In this study, we found that the inhibition of ASIC1a by gene deletion or chemical agents decreased ischemic injury-induced NF-κB activation in vivo and in vitro. Blocking NF-κB with BAY 11-7082 blunted the upregulation of NLRP3 inflammasome components induced by H/R or acidosis. These findings imply that NF-κB participated in ASIC1a-induced activation of the NLRP3 inflammasome. Indeed, a large amount of experimental and clinical data indicate that NF-κB activation plays a vital role in human renal diseases [44]. In the kidney, activating NF-κB in the renal tubular epithelia aggravates tubular injury and exacerbates a maladaptive inflammatory response [49], whereas inhibiting NF-κB reduces renal dysfunction and damage [50]. Normally, NF-κB is sequestered in the cytoplasm in its inactive form. Upon stimulation, NF-κB is released from its inhibitory subunit and is translocated to the nucleus. However, the activation pathways have not been fully identified. Our results showed that H/R or acidic treatment led to the nuclear translocation of NF-κB, which was substantially repressed by blocking ASIC1a in HK-2 cells. These results suggest that activation of ASIC1a by H/R or acidosis promotes the nuclear translocation of NF-κB.

In conclusion, we demonstrated that the NF-κB/NLRP3 inflammasome may be involved in the process of ASIC1a inducing renal IRI. However, the detailed regulatory mechanism and interaction between ASIC1a and NF-κB requires further investigation. ASIC1a inhibition exerted a renoprotective effect (Fig. 7). Thus, ASIC1a may serve as a novel therapeutic target in ischemic AKI.

## Data Availability

Data are available upon request to the corresponding author.
